# A systematic review of person-centered care interventions to improve quality of facility-based delivery

**DOI:** 10.1186/s12978-018-0588-2

**Published:** 2018-10-10

**Authors:** Nicholas Rubashkin, Ruby Warnock, Nadia Diamond-Smith

**Affiliations:** 1 Institute for Global Health Sciences, University of California, San Francisco, Mission Hall, Box 1224, 550 16th Street, Third Floor, San Francisco, CA 94158 USA; 2 Department of Obstetrics, Gynecology & Reproductive Sciences, University of California, San Francisco, USA; 3 Bixby Center for Global Reproductive Health, Department of Obstetrics, Gynecology and Reproductive Sciences, Zuckerberg San Francisco General, University of California, San Francisco, 1001 Potrero Avenue, 6D, San Francisco, CA 94110 USA; 4 Department of Epidemiology and Biostatistics, School of Medicine, University of California, San Francisco, Mission Hall, Box 1224, 550 16th Street, Third Floor, San Francisco, CA 94158 USA

**Keywords:** Systematic review, person-centered care, respectful maternity care, Facility-based childbirth, Interventions, Conceptual frameworks

## Abstract

**Introduction:**

We conducted a systematic review to summarize the global evidence on person-centered care (PCC) interventions in delivery facilities in order to: (1) map the PCC objectives of past interventions (2) to explore the impact of PCC objectives on PCC and clinical outcomes.

**Methods:**

We developed a search strategy based on a current definition of PCC. We searched for English-language, peer-reviewed and original research articles in multiple databases from 1990 to 2016 and conducted hand searches of the Cochrane library and gray literature. We used systematic review methodology that enabled us to extract and synthesize quantitative and qualitative data. We categorized interventions according to their primary and secondary PCC objectives. We categorized outcomes into person-centered and clinical (labor and delivery, perinatal, maternal mental health).

**Results:**

Our initial search strategy yielded 9378 abstracts; we conducted full-text reviews of 32 quantitative, 6 qualitative, 2 mixed-methods studies, and 7 systematic reviews (*N* = 47). Past interventions pursued these primary PCC objectives: autonomy, supportive care, social support, the health facility environment, and dignity. An intervention’s primary and secondary PCC objectives frequently did not align with the measured person-centered outcomes. Generally, PCC interventions either improved or made no difference to person-centered outcomes. There was no clear relationship between PCC objectives and clinical outcomes.

**Conclusions:**

This systematic review presents a comprehensive analysis of facility-based delivery interventions using a current definition of person-centered care. Current definitions of PCC propose new domains of inquiry but may leave out previous domains.

**Electronic supplementary material:**

The online version of this article (10.1186/s12978-018-0588-2) contains supplementary material, which is available to authorized users.

## Plain English summary

When births are conducted in health facilities, it is challenging to balance life-saving surgical interventions, physiologic birth, and humanized care for all women. Person-centered care has recently been proposed as a promising approach to provide evidence-based and equitable birth care that is tailored to a woman’s unique medical and social needs. However, there is no consensus on how to define and implement all or only some aspects of PCC into facility-based delivery settings. Luckily, many past interventions have been designed to improve the level of PCC in birth facilities. We undertook this review of person-centered delivery interventions from 1990 to 2016 in order to understand the full range of past interventions, their person-centered goals, and their impact on clinical and PCC outcomes. We knew that these interventions would be diverse in their designs and goals, so we used a method that allowed us to integrate diverse sources of data.

We used a current definition of person-centered care to systematically search the English-language literature. We explored the relationship between an intervention’s stated person-centered goals and outcomes. We found close to 10,000 abstracts in our original search and narrowed this list to 47 interventions. We found that past interventions principally had the goals to improve the levels of autonomy, supportive care, social support, dignity, as well as the quality of the health facility environment. Past interventions were frequently inconsistent in their stated goals and measured outcomes; in other words, while many interventions intended to impact autonomy, they either did not measure autonomy and/or measured person-centered outcomes unrelated to autonomy. Generally, when researchers measured the level of PCC it either improved or stayed the same. We found no clear relationship between the level of PCC and clinical outcomes.

Our review presents a comprehensive picture of person-centered care interventions conducted in birth facilities. Current definitions of PCC propose new elements that have not been explored well in the past literature. At the same time, past interventions could prove informative around how to enhance PCC through decision-making, continuity midwifery care, and centering in pregnancy.

## Background

In her 1723 impassioned argument against the encroaching class of men-midwives, English midwife Elizabeth Nihell took particular issue with their use of forceps, titling her text *“A treatise on the art of midwifery. Setting forth abuses therein, especially in the practices of instruments* [1]*.”* Possibly the first mention of “abuses” in relation to childbirth in the English-language literature, Nihell foresaw the ongoing debates surrounding obstetric interventions and person-centered care in birth facilities. A spectrum of inappropriate obstetric interventions can be found in today’s birth facilities, from “too much too soon” to “too little too late” [2], with women on both ends of this spectrum experiencing mistreatment [3, 4]. At this time prevalence estimates of mistreatment are challenged by systematic errors in measurement, but nonetheless high percentages of women in many places experience multiple forms of mistreatment during childbirth [5]. Over-medicalization and mistreatment can both lead to excess morbidity and mortality and both represent a violation of women’s fundamental human rights [6, 7].

In developed settings many potential solutions to the problem of over-medicalization and mistreatment of women in birth facilities have been proposed [8–10]. “Person-centered care” (PCC), a concept grounded in strong provider-patient relationships, effective communication and shared-decision making, has figured large in these discussions [11, 12]. Lack of PCC in less developed settings may contribute to delays in care and avoidable maternal mortality [13, 14]. Thus, a person-centered approach holds promise in both developed and less-developed settings to improve quality of maternity care.

### Why this review was necessary

PCC frameworks are complex and feature multiple domains, a fact that may hinder intervention design and result in slow translation of PCC objectives into practice. PCC frameworks span anywhere from 7 to 9 domains of experience [15] and are made up of challenging concepts to operationalize, such as “humanization” and “dignity” [5]. PCC domains could either extensively overlap or be at odds with each other depending on the legal, clinical, or cultural contexts [5]. Finally, there is little to no guidance as to how a given PCC objective, or combination of objectives, might plausibly impact outcomes.

The current complexity of concepts, contexts, and impact when designing PCC interventions could be clarified by using a consistent logic, or theoretical rationale, to inform intervention design. Sales et al. (2006) argue that developing a rationale can “provide a foundation for designing and planning strategies for intervention and selecting tools with a better than random probability of success in implementation [16].” We conducted this systematic review (1) to map the PCC objectives of past interventions using a current definition of PCC delivery care and (2) to explore the impact of PCC objectives on PCC and clinical outcomes.

Refinement of instruments and quantification of the maternity care experience are critical steps prior to development of interventions with a better than random chance of impact. In one recent systematic review Nilver et al. found a wide-ranging set of 36 existing instruments that measure the maternity care experience [17]. Sando et al. found extensive heterogeneity in the sampling techniques, eligibility criteria, and operational definitions of mistreatment during childbirth and advised caution in interpreting prevalence measures of mistreatment [5]. Adding to the complexity, existing studies span countries and regions, and there may be cultural differences in how women define positive person-centered experiences, not to mention the diversity of both biomedical and midwifery models in health systems around the world [18]. Similar to these reviews, we expected to find an expansive literature on the subject of person-centered care in birth facilities. Our paper uses a review method that is particularly suited to synthesizing disparate literatures and is a timely contribution to those seeking to design impactful interventions to improve the quality of maternity care around the world.

### Person-Centered Care Frameworks in the Global Context

Person-centered delivery care in developed settings emerged almost exclusively in response to the impersonal and excessive medicalization of childbirth [2]. Only recently has PCC become an area of inquiry as a potential deterrent to facility-based childbirth [3]. Modern objections to the over-medicalization of childbirth are rooted in events of the early twentieth century when white women in Europe and America began to pursue ‘twilight sleep’ [19]. The woman’s movement of the 1960s and 1970s redefined birth into an event of social and personal significance, ideally controlled by an awake and empowered mother [20–22]. Around the same time midwifery care re-emerged and became solidified to different extents in the official health systems of Europe and North America [23].

By the early 1990s efforts to reform the experience of maternity care and to shore up the profession of midwifery in developed nations were fully underway. A direct line between current definitions of PCC delivery care can be drawn to the United Kingdom’s National Health Service’s 1993 *Changing Childbirth* expert report. The report included both a strategy for midwifery care and a “Patient’s Charter” that laid out the rights of maternity patients [24]. Meanwhile, in the international context the 1994 International Conference on Population and Development in Cairo became a turning point for a rights-based approach to sexual and reproductive health [25]. Twenty years later a synthesis of international reproductive rights declarations produced the first publications on “respectful maternity care” (RMC) [3, 26]. The RMC frameworks were soon followed by statements from all the major international health organizations denouncing the mistreatment of women in childbirth [4]. The WHO, leveraging its power as a norm-setting organization, then published a framework to establish the experience of care as a pillar of quality maternity care [27].

Now with close to 30 years of discussion around person-centered care, several overlapping strains of PCC exist, but with gaps between the different approaches. We described one strain above that aligns with the re-emergence of midwifery care and includes different approaches to the provision of care (continuity midwifery models, centering in pregnancy, doula-supported childbirth). More recently, the Lancet Midwifery series examined the contributions of midwives in the global context, including resource-poor settings which have not been exposed to excessive medicalization [28]. A second strain of PCC in the global literature has emerged relatively separate from midwifery, namely the framework around mistreatment of pregnant women, as advanced by Bowser and Hill [3]. Bohren et al. revised the Bowser and Hill typology to develop the most comprehensive set of PCC categories in the maternity context [15]. However, between the midwifery approach and the mistreatment typologies there existed a persistent gap as to how these two areas might be inter-related.

Thus, a broader approach was necessary if the model of care and mistreatment categories were going to be useful in a range of resource settings and in health systems with different proportions of technological obstetric care and primary midwifery care. Given this tension between local contexts and universal frameworks for PCC and for models of care, Sudhinaraset et al. [29] conducted a trans-disciplinary review to create the most comprehensive and adaptable PCC framework to date, which they call The Person-Centered Framework for Reproductive Health Equity. In this framework Sudhinaraset et al. link the provision of care to the experience of person-centered care, using a similar typology to Bohren et al. However, they go beyond the typology approach and link the provision of care to PCC. The provision of care encompasses evidence-based care, both the over and underuse of technology, information and referral systems, infrastructure, human resources, and the medical supply chain. Sudhinaraset and colleagues also situate PCC within the context of a community’s experiences with care, as a community’s specific history with discrimination can determine care-seeking behaviors. Ongoing experiences with mistreatment in the facility can in turn influence a community’s care-seeking behaviors. Finally, the authors link the facility and care- seeking behaviors to societal and community determinants of health equity, including gender and violence norms [30, 31]. In this review, we chose to use Sudhinaraset and colleagues’ framework as we see theirs as the most comprehensive and flexible PCC framework. Importantly, for a global review of PCC delivery interventions, the Sudhinaraset et al. framework points to the ways in which PCC components need to be contextualized within specific health systems, gender and violence norms, and community behaviors [29]. Finally, this systematic review protocol and the PCC Framework for Reproductive Health Equity were developed contemporaneously within a cooperating research group.

## Methodology

We knew that the interventions and outcomes included under the framework of person-centered care would not be amenable to meta-analysis. As a result, this systematic review applied the qualitative method of framework analysis in order to define concepts, map the range of the phenomena, create typologies, find associations, seek explanations, and develop new ideas [32]. The initial step consisted of a systematic approach to problem identification, which we identified to be the complexity of concepts, contexts, and potential impacts that result from PCC frameworks. We followed reporting standards for systematic reviews of social interventions set forth by the Campbell Collaboration [33], including the development and publication of a protocol with pre-determined inclusion criteria and analysis plan which was registered with the PROSPERO International prospective register of systematic reviews [34].

### Inclusion and exclusion criteria

In order to be included, an article had to: (1) contain original data (quantitative or qualitative), (2) consist of an evaluation, (3) have at least one PCC objective designed into the intervention and (4) be facility-based. We defined quantitative data as using inferential statistics and qualitative data as primary narratives from participants. We defined an “evaluation” as any quantitative study that utilized a control group (experimental, quasi-experimental). Quasi-experimental quantitative studies needed to collect longitudinal and/or cross-sectional data from treatment and comparison groups. A qualitative evaluation had to be associated with a new person-centered delivery intervention, but we did not require a control group. We defined an objective as the primary goal that the intervention sought to impact. We defined “person-centered” objectives using a current definition grounded in the literature, encompassing: dignity, autonomy, privacy/confidentiality, communication, social support, supportive care, trust, and the health facility environment (See Additional file 1: Table S1 for definitions) [29]. We defined “facility-based” as having some linkage to a hospital or birth center and, required that outcomes be measured at the level of the facility. We defined person-centered outcomes according to the same criteria as the objectives. We defined clinical outcomes to include: labor and delivery, perinatal, and maternal mental health. If a PCC intervention was conducted in the prenatal setting, the measured outcomes had to cross over into the delivery setting.

We excluded quantitative studies that lacked a valid control or comparison group, exclusively examined prenatal outcomes (e.g., ambulatory prenatal diabetes care) or postpartum outcomes (e.g., breastfeeding). We recorded the number of excluded studies and the reason for exclusion at each stage.

### Search strategy

We designed a search strategy to maximize the number of primary sources. We searched the English language literature from 1990 until 2016. We conducted a first search in October 2015 and a second search in April 2016 in order to identify any new publications. We systematically searched peer-reviewed literature in PubMed, CINAHL, EconLit, and EMBASE using controlled search terms and free-text terms combining three main components: (a) pregnancy and delivery care (b) person-centered care and (c) interventions (See Additional file 2: Search Strategy). The final keyword chain from April 2016 differed slightly in that we added terms for group prenatal care and birth plans. Otherwise, the 2016 search was only adjusted for the date of publication. The same keywords were used in CINAHL, EconLit, and EMBASE according to their respective search engine requirements.

We hand searched the Cochrane database for all studies related to maternity care. We extensively searched the gray literature, including reports from relevant governmental and non-governmental organizations’ websites by using Google Scholar keyword searches (See Additional file 2: Search Strategy). We searched dissertations and theses in the ProQuest database. Finally, we used bibliographic back referencing to identify additional studies not captured by any of the above searches. We maintained a search diary describing the search methods, keywords used, and search results.

### Screening and data extraction

We excluded duplicate references. Next, we independently reviewed titles, abstracts, and executive summaries; we excluded all references that were clearly not relevant. Following this, two team members independently applied the pre-specified inclusion/exclusion criteria to the remaining abstracts. Disagreements regarding the inclusion status of any article were presented to a third team member for a final decision. When the abstract did not contain sufficient information for inclusion, the full text was retrieved.

Three researchers then independently performed full-text reviews and extracted quantitative or qualitative data. Data extractions were checked by one other researcher. Descriptions of interventions were assembled including the study setting; sample characteristics; objectives; design; data collection and analysis methods. We extracted person-centered and clinical outcomes that had significance testing of *p* < 0.05. We summarized these outcomes in tables in a qualitative manner by the direction of their effect (positive, negative, no differences). Two data extractors worked independently, followed by an independent third checker. Themes, findings, and participant quotations were extracted from qualitative studies.

### Analysis

We first categorized interventions according to their PCC objectives. All interventions were assigned a primary PCC objective, such as “autonomy” or “supportive care,” through a consensus process. An intervention frequently was assigned multiple secondary PCC objectives. Then, undergoing a data reduction process, interventions that shared a primary objective were grouped together and then sub-categorized into conceptual groupings. Following Sudhinaraset et al.’s framework, we formed the conceptual groupings into established models of care.

We categorized outcomes using the same categories as the PCC objectives. We summarized overarching themes and directions of PCC and clinical outcomes. We conducted a thematic analysis of qualitative data [35], a process that included reading repeatedly to extract concepts, categories, and metaphors used to describe or interpret PCC as experienced by the women interviewed. We integrated the quantitative and qualitative data into a final summary. We restricted our search terms by intervention type, rather than outcomes and thus included studies that measured a range from outcomes, from clinical maternal and perinatal outcomes to satisfaction and mental health. Given the diversity of quantitative outcomes, we are not able to combine results to make statements about effect sizes.

### Data quality assessment and risk of bias

Two researchers independently assessed the risk of selection, confounding, performance, and reporting bias; we coded the studies as low, medium, or high risk for each of the four types of bias. Qualitative data were independently appraised by two researchers using the 9-item Critical Appraisal Skills Programme Qualitative Research Checklist [36]. Risk of bias and quality assessment summaries can be found in the Additional file 3: Figure S1 and Table S2.

## Results

### General overview

**Fig. 1:**
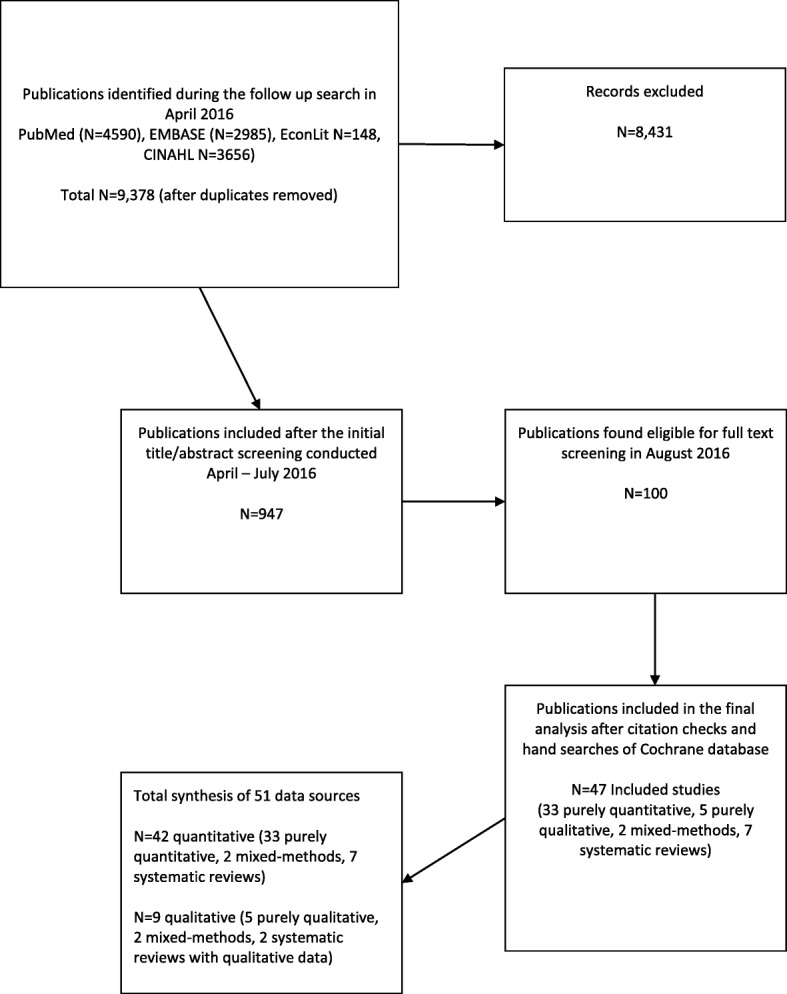
Study search flow diagram

The initial database searches yielded 11,409 articles with *N* = 9378 after duplicates were removed. After title-abstract screening was performed, *N* = 947 remained, and after full text reviews *N* = 100 potentially eligible studies were found. Of the final included studies (*N* = 47), 34 resulted from database searches and were supplemented by hand-searches (*N* = 7), bibliographic back referencing (*n* = 5), and theses and dissertations (*n* = 1). The review process and descriptive characteristics of the studies are reviewed in Fig. 1 and Table 1.

**Table 1:** Characteristics of all articles included in the review

Characteristic of the publication	Number of publications (%)	Publication reference list
Year of publication		
1990–1999	5/47	[39, 42, 47, 73, 74]
2000–2009	19/47	
2010–2016	23/47	[37, 44, 46, 49–54, 58, 61, 63, 66, 65–67, 69, 71, 79–84] [41, 42, 44, 45, 48, 51, 56, 60, 71, 73]
Location of the study		
Africa	2/47	[60, 71]
Australia	4/47	[43, 52, 56, 58]
East Asia	3/47	[37, 46, 77]
Europe	9/47	[38, 45, 47, 57, 73, 75, 76, 81, 82]
Latin America	1/47	[79]
Middle East	2/47	[40, 59]
North America	18/47	[39, 42, 48–51, 54, 55, 63, 64, 66–68, 70, 74, 78, 80, 83]
South Asia	1/47	[62]
Multiple locations (systematic reviews)	7/47	[8, 41, 44, 53, 61, 65, 69]
Sample Size		
< 30	4/47	[47, 66–68]
30–99	9/47	[39, 43, 58, 59, 74, 78–80, 82]
100–499	14/47	[37, 38, 40, 46, 49, 57, 62–64, 70, 75, 77, 81, 83]
500–999	3/47	[46, 64, 80]
1000–4999	11/47	[41, 42, 44, 45, 48, 51, 56, 60, 65, 71, 73]
> 5000	6/47	[53–55, 61, 69, 84]

We categorized interventions (both quantitative and qualitative findings) into five primary PCC objectives: autonomy (*N* = 20), supportive care (*N* = 17), social support (*N* = 11), the health facility environment (*N* = 2), and dignity (*N* = 1). We did not find any intervention designed with these primary objectives: trust, privacy/confidentiality, or communication. Nonetheless, these three domains were addressed as secondary objectives. Below brief summaries of the results are organized from most common primary PCC objective to least common (for definitions of PCC objectives/outcomes and more detailed descriptions of the interventions, see Additional file 1: Table S1). In Table 2 we present detailed information about each quantitative study, PCC domain, intervention type and description, and the effect size of the outcomes; Table 3 presents the comparable information for the qualitative studies. In Tables 5, 6, 7 and 8 we briefly summarize the quantitative interventions and in Table 9 the qualitative interventions. Each table (Tables 5, 6, 7, 8 and 9) represents a single primary PCC objective; most tables contain a sub-grouping of interventions according to a model of care (for instance, “continuity midwifery” under the PCC objective of “autonomy”). Each intervention has two rows: the first row contains the PCC objectives (marked with an “X”); the second row contains PCC and clinical outcomes. The key below explains how to interpret the direction of the outcomes.

**Table 2:** Detailed descriptions of included quantitative studies, organized by primary PCC objective

	Author and title	Type of intervention	Intervention details	Outcomes (Person-centered care (PCC), labor and delivery, perinatal, mental health)
Person-Centered Objective: Autonomy				
1	Benjamin, 2001	Autonomy	Intervention: Continuity midwifery model consisting of a pair of midwives providing care to one woman through prenatal, birth, and postpartum. Where: United Kingdom Population: Pregnant Women Study design: Prospective, non-randomized clinical trial Sample size: 611	PCC: attended in birth by a known midwife (OR 39.65, *p* < 0.001). Labor and delivery: higher home birth (OR 15.38, *p* < 0.001), lower epidural (OR 0.56, *p* = 0.002), higher upright birth (OR 9.64, *p* < 0.001), higher intact perineum (1.57, *p* = 0.027), higher physiologic third stage (OR 38.69, *p* < 0.001), lower induction of labor (OR 0.66, *p* = 0.042). Perinatal: No significant difference in Apgar scores, Admission to Neonatal Unit (NNU) and death.
2.	Brown, 2015	Autonomy	Systematic Review of interventions that gave women their own case notes to carry in pregnancy, 4 trials included. Sample Size: 1176	PCC: Women felt more in control (RR 1.56, 95% CI 1.18 to 2.06), no difference in satisfaction. Labor and delivery: More women had operative deliveries (RR 1.83, 95% CI 1.08 to 3.12), and caesarean sections (RR 1.51, 95% CI 1.10 to 2.08), no difference in analgesics. Perinatal: No difference in stillbirth. Mental Health: No difference in maternal depression.
3.	De Koninck, 2001	Autonomy	Intervention: Continuity midwifery model implemented into birth centers that employed 3–6 midwives to provide care to one woman through prenatal, birth, and postpartum. Where: Canada Population: Pregnant women Study design: Intervention and matched controls Sample size: 2000	PCC: Longer visits (78 vs. 33 min, *p* < 0.001), had the opportunity to ask questions “very often” (84.6% vs. 64.1%, *p* < 0.001), rated their care as “very personalized” (87.9% vs. 33% *p* < 0.001). Delivered by a continuity provider (70.5% vs. 38.8%), able to choose labor position (84% vs. 25%, *p* < 0.001). Feeling of control over delivery (mean 4.33 vs. 3.95, p *p* < 0.001).
4.	Fraser, 1997	Autonomy	Intervention: Prenatal education and support given by a research nurse coordinator. Where: Canada Population: Women with a prior cesarean Study design: Randomized controlled trail Sample size: 21	PCC: No difference in perception of control on the Birth Experience Rating Scale. Labor and delivery: No difference in vaginal delivery. Perinatal: No differences in perinatal mortality or maternal morbidity.
5.	Gerancher, 2000	Autonomy	Intervention: Verbal consent process for epidural anesthesia with a written consent form, reviewed and signed by both the patient and the investigator, patient received copy of the written consent form for their reference. Where: United States Population: Women in labor Study design: Randomized to intervention Sample size: 82	PCC: Better recall scores of information in the written and verbal consent group (*p* < 0.001).
6.	Gu, 2013	Autonomy	Intervention: A new midwife antenatal clinic (not a continuity model because the midwives did not provide intrapartum care). Where: China Population: Primiparous pregnant women Study design: Randomized controlled trial Sample size: 110	PCC: More satisfaction upon admission (*p* < 0.001) and more satisfaction with the perinatal care experiences (*p* < 0.001). Labor and Delivery: Higher vaginal delivery (66% vs. 43%, 95% CI 3.69–41.60). No significant differences in mean maternal blood loss. Perinatal: No significant differences in Apgar scores. Mental Health: No difference in anxiety.
7.	Horey, 2004	Autonomy	Systematic review of interventions to support women’s decision-making about mode of birth after cesarean. Three Randomized controlled trials were included. Sample size: 2270	PCC: Less decision conflict about preferred mode of birth (SMD −0.25; 95% CI -0.47 to − 0.02); no increase in knowledge with decision support; no difference in satisfaction. Labor and delivery: No significant difference in vaginal birth, elective/scheduled caesarean and attempted vaginal delivery. Perinatal: no significant differences in adverse outcomes.
8.	Kuo, 2010	Autonomy	Intervention: A birth plan that consisted of a detailed conversation with a nurse about common procedures encountered on labor and delivery, women then signed an individualized birth plan with their obstetrician. Where: Taiwan Population: Pregnant women, no complications Study design: A randomized, single-blind controlled trial Sample size: 296	PCC: More positive childbirth experiences (*t* = 2.48, *p* = 0.01), higher degree of childbirth control (*t* = 9.60, *p* < 0.001), no difference in prenatal childbirth expectations; higher postnatal fulfillment of childbirth expectations after delivery (*t* = 2.63, *p* = 0.01), especially mastery and participation subscale (*t* = 3.74, *p* = 0.001). No difference in care-giving environment, spousal support, labor pain expectations, or medical support.
9.	Lundgren, 2003	Autonomy	Intervention: Antepartum questionnaire and a birth plan formulation. Where: Sweden Population: Women not planning elective caesarean section Study Design: All women in a set period of time were invited to participate, compared to women in same facilities in period directly before. Sample size: 271	PCC: Lower scores for the relationship to the first midwife they met during delivery (*p* < 0.05, domains: listening and paying attention to needs and desires, support, guiding, and respect). No difference with time spent, competence, trust, or support. Labor and Delivery: No difference in fear of childbirth, pain during childbirth, sense of control, concerns for the child, and the total experience.
10.	Macfarlane, 2014	Autonomy	Intervention: A new freestanding birth center. Where: United Kingdom Population: Women living in a low socio-economic Inner- city area Study design: Pre/Post evaluation Sample size: 620	PCC: More choice for birthing position (83.8% vs. 51.6%); told to follow their own urge to push (52.2% vs. 16.9%). Women reported 29.7% higher satisfaction (good and very good care) overall 95%CI, −38.5, −18.7 and reported staff were always kind and understanding 38.2 95%CI, −47.7, −27.4. More women were cared for my a midwife they had already met 37.9%, 95%CI, − 49.5, −25.8, had one and one care all the time 36.9%, 95%CI, − 47.9, − 23.6. More women used a birth plan 19.5 95%CI, − 33.0, − 4.8. Women reported greater privacy (always) 19%, 95%CI, − 28.9, − 8.1, respect and dignity (yes, definitely) 34.8% -44.6, − 23.8, cleanliness (Yes, very clean) 56.2%, 95%CI, − 65.6, − 44.0. Labor and Delivery: fewer inductions (10% vs. 20.2), fewer AROM (13.3% vs 26.7%), more ability to move in labor (92% vs. 70.5%), more spontaneous vaginal birth (73.8% vs. 62.2%), fewer episiotomy (11.1% vs. 17.0%). No significant differences in oxytocin augmentation or continuous electronic fetal monitoring (EFM).
11.	Martin, 2014	Autonomy	Intervention: A specialty clinic for women who experienced a prior caesarean, designed to create a supportive environment in order to address childbirth fear, confidence, and knowledge and intention to pursue a Vaginal Birth After Cesarean (VBAC) in the current pregnancy. Where: Australia Population: Women with a prior caesarean Study Design: Comparative descriptive study Sample size: 92	PCC: More knowledge of behavioral techniques to cope with labor and birth (81.8% vs 50%); no significant change over time within or between groups in childbirth fear; increase in childbirth self-efficacy at 36 weeks GA (*p* = 0.01). Higher preference for VBAC at 36 weeks GA (80% vs. 56.3%). Labor and delivery: No difference in actual VBAC rates.
12.	Martinez, 1992	Autonomy	Intervention: Early Intrapartal Childbirth Preparation included labor information and practice strategies, in a twenty-minute session during the latent phase of labor. Where: United States Population: Women in labor Study Design: Random assignment to study group Sample size: 89	Labor and delivery: Shorter Stage 1 of labor; higher holism associated with decreased length of labor. Mental Health: No differences on emotional response to labor. Higher coherence associated with less negative emotional responses for all subjects.
13.	McCourt, 1988	Autonomy	Intervention: One-to-one midwifery care practice where one midwife plans and provides the majority of antenatal, intrapartum, and postpartum care. Where: United Kingdom Population: Pregnant women Study Design: Prospective, all women in intervention facilities compared to control facilities in different postal area Sample size: 1400	PCC: More likely to have named midwife as primary caregiver (97% vs 74%), to say they knew their primary provider “very well” (16% vs 4%), preferred to see their primary caregiver (86% vs 50%), to state they were “very well prepared” for birth (18% vs 12%), to feel confident about labor (51% vs 39%), to rate the birth as “hard work but wonderful” (51% vs 39%), have continuous support from midwife (90% vs 53%), and more likely to be “very satisfied” (79% vs 71%). No differences in listening or explanations. Labor and Delivery: fewer augmentations of labor (29% vs. 37%).
14.	Mehdizade, 2005	Autonomy	Intervention: Birth preparation classes including pedagogic material, counseling sessions, and neuromuscular exercises. Where: Iran Population: Primigravid women under 35 Study Design: Random assignment to intervention and control groups. Sample size: 200	Labor and delivery: Lower rate of caesarean section (*p* = 0.044), shorter length of labor (*p* = 0.0016), more use of oxytocin (*p* = 0.033), less back/pelvic pain (*p* = 0.0043 two sided t test), more headache (*p* = 0.015), less disturbed sleep (*p* = 0.085). No difference in analgesic/epidural use or episiotomy. Perinatal outcomes: No difference in newborn weight or Apgar score.
15.	O’Cathain, 2002	Autonomy	Intervention: 10 pairs of informed choice leaflets covering prenatal health and labor topics. Where: United Kingdom (Wales) Population: Pregnant women Study Design: Cluster trial, with maternity units randomized to intervention and control Sample size: 6452	PCC: Increase in satisfaction with information (OR = 1.4), no difference in: women reporting that they exercised informed choice, active decision making, support of partner. Labor and delivery: No difference in planned place of birth, epidural use, in staying in bed during labor. Mental health: No difference in anxiety.
16.	Sandall, 2015	Autonomy	Systematic review and meta-analysis of Midwife-led continuity models versus other models of care. Fifteen randomized controlled trials included. Sample size: 17,674	PCC (selected): Dignity (Midwife interested in me as a person, OR 7.50); Autonomy (multiple measures higher for satisfaction, decision making); Communication (asking questions *t* = 6.6; encouraged to ask question OR 4.22); Supportive care (midwives always friendly, OR 3.48); Trust (midwife skill *t* = 3.44). Labor and Delivery: Fewer epidurals (0.85, 95%CI 0.78 to 0.92), fewer instrumental vaginal delivery (RR 0.90, 95%CI 0.83 to 0.97), more spontaneous vaginal delivery (RR 1.05, 95%CI 1.03 to 1.07). No differences in caesarean section or intact perineum. Perinatal: Fewer preterm births (RR 0.76, 95%CI 0.64–0.91), fewer neonatal deaths (RR 0.84, 95%CI 0.71 to 0.99).
Person-centered Objective: Supportive Care				
17.	Consonni, 2010	Supportive Care	Intervention: Ten prenatal meetings with these elements: educational (pregnancy knowledge), physiotherapeutic (breathing, kinesiotherapy, relaxation), interaction components (discussing pregnancy experiences, emotions), and relaxation (physical and mental). Where: Brazil Population: Nulliparous pregnant women Study design: Not randomized controlled trial, group selection based on participation Sample size: 67	Labor and delivery: More vaginal birth (81% vs. 58.6%, *p* < 0.05 chi square test). Perinatal: No difference in preterm birth, birth weight or Apgar < 7 at 5 min. Mental health: Lower trace anxiety (*p* < 0.05 independent t-test).
18.	El-Mohandes, 2011	Supportive Care	Intervention: Integrated behavioral intervention based on social cognitive theory. Where: United States Population: High risk African-American pregnant women Study design: randomized controlled trial, intent-to-treat analysis Sample size: 819	Perinatal: Fewer very preterm births (OR = 0.42, 95% CI = 0.19–0.93) (not significant for low birth weight (LBW) or preterm). Mental Health: No difference in depression scale.
19.	Gagnon, 1999	Supportive Care	Intervention: One-to-one nursing care, which consisted of emotional and physical support for women undergoing oxytocin labor augmentation. Where: United States Population: Pregnant women, singleton Study Design: Secondary analysis of a randomized controlled trial Sample size: 100	Labor and delivery: No significant differences in cesarean delivery, epidural anesthesia, instrumental delivery, intact perineum, or mean duration of labor. Perinatal: No difference in Neonatal Intensive Care Unit (NICU) admission.
20.	Grassley, 2012	Supportive Care	Intervention: Four maternity care visits by Intrapartum nurses and professional labor support by attending to physical and emotional needs. Where: United States Population: Pregnant adolescents Study Design: Separate sample posttest quasi-experimental Sample size: 106	PCC: Higher scores on the Mackey Childbirth Satisfaction Rating Scale (*p* = 0.02). Labor and Delivery: No difference in vaginal delivery.
21.	Harris, 2012	Supportive Care	Intervention: Interdisciplinary program to promote physiologic birth and encourage active involvement of women and their families in maternity care. Where: Canada Population: Low income pregnant women Study design: Retrospective chart review of intervention facility compared to women in non-intervention facilities Sample size: 1238	Labor and Delivery: More likely to plan a VBAC (RR 3.22, 95%CI 2.25–4.62), to be delivered by a midwife (41.9% vs. 7.4%, *p* < 0.001), to have intermittent fetal auscultation (RR 1.41, 95%CI 1.31–1.53), to have a 3rd degree laceration ((RR 1.23, 95%CI 1.08–1.40). Less likely to have an epidural (RR 0.75, 95%CI 0.69–0.81), to undergo induction of labor (RR0.83, 95%CI 0.74–0.93), to undergo cesarean section (RR 0.76, 95%CI 0.68–0.84). No difference in assisted vaginal delivery. Perinatal: Higher gestational age at delivery (39.2 vs 38.8, *p* < 0.0001), birth weight (3395.3 vs. 3315.9, *p* < 0.0001). No difference in stillbirth, Apgar< 7 at 5 min, or NICU admission.
22.	Hodnett, 2010	Supportive Care	Systematic review of interventions that provided additional support for women believed to be at high risk of low birth weight. Seventeen trials included. Sample size: 15,288	PCC: No difference in satisfaction. Labor and delivery: Reduction in caesarean section (RR 0.87, 95% CI 0.78 to 0.97) Perinatal outcomes: No effect on preterm birth, LBW, or stillbirth. Mental Health: No difference in postpartum depression.
23.	Ip, 2009	Supportive Care	Intervention: Enhanced women’s self-efficacy for childbirth and coping abilities for pain and anxiety through two 90-min educational sessions. Where: China Population: Primigravidae pregnant women Study Design: Randomized controlled trial Sample size: 133	PCC: Higher levels of self-efficacy for childbirth (*p* < 0.0001), and greater performance of coping behavior during labor (*p* < 0.01). Labor and Delivery: Lower perceived anxiety (*p* < 0.001, early stage and *p* = 0.02, middle stage) and pain (*p* < 0.01, early stage and *p* = 0.01, middle stage). Mental Health: Lower perceived anxiety (*p* < 0.001, early stage and *p* = 0.02, middle stage).
24.	Kildea, 2012	Supportive Care	Intervention: A specialist antenatal clinic using participatory methods. Where: Australia Population: Indigenous (Aboriginal and Torres Strait Islander) Australian pregnant women Study Design: Women who attended specialist clinic compared to women in same facility and time period who did not Sample size: 800	PCC: One-question for culturally responsive care “Felt most understood” at the specialty clinic (92%) vs. birth suite (47%). Labor and Delivery: Increased prenatal visits (*p* = 0.007), more spontaneous vaginal births (*p* = 0.06), more intact perineum (*p* < 0.001). No differences in analgesia, and postpartum bleeding. Perinatal outcomes: No differences in preterm birth, 5 min Apgar < 7, LBW, NICU admission.
25.	Mason, 2011	Supportive Care	Intervention: A case management program, to improve prenatal and post-partum care through enhanced member outreach and incentives, wellness materials, intensive case management, and provider incentives. Where: United States Population: Medicaid recipients Study Design: Retrospective propensity adjusted cohort comparison Sample size: 76735	Perinatal outcomes: LBW less likely to have poor outcome (OR 0.921, 95%CI 0.869–0.975).
26.	Newman, 2008	Supportive Care	Intervention: Prevention of Preterm Birth (PTB) through case identification, risk assessment, 24 h perinatal hotline, high risk case management. Where: United States Population: Medicaid population with any of 9 predetermined historical or current pregnancy high-risk triggers Study Design: Pre/post design Sample size: 6356	Perinatal outcomes: Reduction in PTB below 28 weeks (RR 0.75, 95%CI 0.5–0.96 *p* = 0.029), reduction in frequency (RR 0.86, 95%CI 0.75–0.98) *p* = 0.04) and mean duration of NICU admission (25.0 vs 20.6 days, *p* = 0.01).
27.	Panaretto, 2005	Supportive Care	Intervention: A collaborative prenatal care program for women based on common sense, continuity of care, cultural currency and a family-friendly environment, cultural safety aspects of the Aboriginal Medical Service and the collocation of mental health, dental and social support services. Where: Australia Population: Indigenous, urban women Study Design: Pre/Post evaluation Sample size: 1000	Labor and Delivery: Increased number of prenatal visit (3 vs. 7, *p* < 0.001). Perinatal outcomes: Fewer preterm births (8.7% vs 14.3%, *p* < 0.01). No difference in LBW or perinatal mortality.
28.	Rouhe, 2013	Supportive Care	Intervention: Intervention for women with severe fear of childbirth with six sessions of psycho-educative group therapy led by a continuity psychologist, including a guided relaxation exercise. Where: Finland Population: nulliparous women with fear of childbirth Study design: randomized controlled trial Sample size: 400	PCC: Higher positive delivery experience > 75 centile on delivery satisfaction scale (DSS) scale (36.1 vs. 22.8%, *p* = 0.04), and lower Wijma Delivery Experience Questionnaire (W-DEQ-B) scores 63.0 vs. 73.7, *p* = 0.02). Labor and delivery: More spontaneous vaginal births (63% vs. 47% *p* = 0.005) and fewer caesarean section (22.9% vs. 32.5%, *p* = 0.05). No difference in epidural, induction of labor, length of labor. Perinatal outcomes: No difference in birth weight, cord artery pH < 7.1, 1 min Apgar < 7.
29.	Ryding, 2003	Supportive Care	Intervention: Consultation with specially trained midwives, including discussion about past traumatic experiences (birth or childhood) and to development of a birth plan. Where: Sweden Population: Women with fear of childbirth Study Design: Women who consulted midwives for fear of childbirth and got intervention matched to women in same facility who did not receive intervention Sample size: 112	PCC: Higher negative/frightening experience (W-DEQ mean difference 14.6, *p =* 0.0001). Labor and delivery: More vaginal delivery (44.7% vs 27.5%). Mental health: Higher Impact of Event Scale (IES) score > 30 indicating possible Post-Traumatic Stress Disorder (PTSD) (19% vs 2%, OR 12.1, 95%CI 2.2–66.6).
30.	Saisto, 2001	Supportive Care	Intervention: Intensive therapy group for fear of childbirth, including discussion of obstetric experiences, feelings, misconceptions. The therapy was integrated into routine antenatal care and combined with cognitive exercises. Where: Finland Population: Pregnant women with fear of childbirth Study Design: A Randomized Controlled Trial Sample size: 176	PCC: Decrease in birth related concerns (*p* = 0.022). No difference in satisfaction with childbirth or in puerperal depression. More intervention women remembered, “not feeling safe” (*p* = 0.02). Labor and delivery: Fewer maternal request cesareans (36% vs 41% of original request, *p* > 0.05) and shorter labor (6.8 h vs 8.5 h, *p* = 0.039) Mental health: Decrease in pregnancy-related anxiety (*p* = 0.054). No difference in depression.
31.	Vieten, 2008	Supportive Care	Intervention: A Mindful Motherhood intervention including general mindfulness strategies such as awareness of thoughts and feelings, guided body awareness and yoga, and acceptance of self. This also included awareness of the developing fetus, mindfulness around pregnancy/labor pain and parenting, and prenatal yoga. Where: United States Population: Pregnant women with “mood concerns” Study design: randomized trial Sample size: 21	Mental Health: Greater % improvement at 8 weeks post intervention for anxiety, depression, perceived stress, positive affect, negative affect, mindfulness, and affect regulation. However, these changes were diminished at 3-month follow up.
Person-Centered Objective: Social support				
32.	Barr, 2011	Social Support	Intervention: Group prenatal care model implemented into a family practice residency program. Where: United States Population: Pregnant women Study Design: Pre- and post-intervention design Sample size: 400	Labor and Delivery: Lower odds of cesarean (OR 0.61, 95%CI 0.37–1.01). Perinatal outcomes: Lower LBW (OR 0.43, 95%CI 0.18–1.06) and preterm birth (OR 0.39, 95%CI 0.15–0.98).
33.	Bloom, 2005	Social Support	Intervention: Group antenatal care (ANC) provided by midwives for adolescents in a public school setting Where: United States Population: Pregnant Adolescents Study Design: Intervention compared to adolescents receiving standard ANC care Sample size: 120	PCC: Improvement in knowledge (100% Group ANC vs. 55% control, *p* < 0.05). No significant differences with self-esteem or health locus of control. Perinatal: No significant difference in preterm births.
34.	Catling, 2015	Social Support	Systematic review and meta-analysis of group vs. conventional ANC. Four group antenatal care randomized controlled trails. Sample size: 2350	PCC: marginally higher satisfaction (mean diff 4.90, 95%CI 3.10–6.70, *p* < 0.001). No differences in perceived stress. Labor and delivery: No significant differences in induction/augmentation of labor, epidural use, episiotomy, or spontaneous vaginal birth. Perinatal: No significant differences in preterm birth, LBW, SGA, perinatal mortality. Mental health: No differences in depression.
35.	Gruber, 2013	Social Support	Intervention: women were given the option of a having a doula or not. Where: United States Population: Socially disadvantaged pregnant women Study design: Non-experimental design with assignment to groups (doula vs. non-doula) based on self selection Sample size: 226	Labor and delivery: No difference in vaginal delivery or maternal complications. Perinatal outcomes: Fewer lower birth weight babies (z score = 1.78, *p* = .04).
36.	Gungor, 2007	Social Support	Intervention: Fathers allowed in labor room, oriented to delivery room and birth process, allowed to be present in delivery. Where: Turkey Population: Primigravidae low-risk pregnant women who wanted their partner to be present Study Design: First half of eligible women received intervention compared to the second half of eligible women Sample size: 50	PCC: More positive view of delivery process, labor process, partner participation, awareness and delivery outcome (*p* < 0.05 for all). Labor and delivery: no difference in pain medication, use of obstetric interventions, or labor length.
37.	Hodnett, 2013	Social Support	Systematic Review of interventions on continuous support compared to standard care. Twenty-two studies included. Sample size: 12,264	PCC: Less likely to report dissatisfaction (RR 0.69, 95% CI 0.59–0.79). Labor and delivery: More spontaneous vaginal birth (RR 1.08, 95%CI 1.04–1.12), less intrapartum analgesia (RR 0.90, 95% CI 0.84–0.96) and regional analgesia (RR 0.93, 95% CI 0.88–0.99), shorter labors (MD −0.58 h, 95% CI -0.85 - 0.31), less likely to have a caesarean (RR 0.78, 95% CI 0.67–0.91) or instrumental vaginal birth (fixed-effect, RR 0.90, 95% CI 0.85–0.96). No difference on maternal complications. Perinatal outcomes: Lower risk of baby with low five-minute Apgar score (fixed-effect, RR 0.69, 95% CI 0.50–0.95). No difference on neonatal complications.
38.	Kunene, 2004	Social Support	Intervention: Providing training to health providers on couple counseling, invited partners of antenatal women to attend counseling twice during pregnancy and once post-delivery, and provided information to couples. Where: South Africa Population: Pregnant women and partners Study Design: Cluster randomized controlled trial Sample size: 2082	PCC: Partner more likely to assist during pregnancy emergencies (*p* = 0.004).
39.	Mullany, 2007	Social Support	Intervention: Husband present for pregnancy health education visits, consisting of two 35-min sessions based on the principals of reasoned action and the health belief model. Where: Nepal Population: Pregnant women Study Design: Randomization Sample size: 442	PCC: More likely to make > 3 birth preparations (RR 1.99, 95%CI 1.10–3.59). Labor and Delivery: No difference in attending prenatal visits, delivering in an institution, or having a skilled provider at birth.
Person-centered Objective: The care environment				
40.	Hodnett, 2012	The Care Environment	Systematic review and meta-analysis of alternative institutional birth settings. Ten studies included. Sample size: 11,795	PCC: Increased “very positive” views of care (RR 1.96, 95%CI 1.78–2.15). Labor and Delivery: Decreased epidural anesthesia (RR 0.8, 95%CI 0.74–0.87), decreased oxytocin augmentation 0.77, 95%CI 0.67–0.88), increased vaginal birth (RR 1.03, 95%CI 1.02–1.06), decreased episiotomy (RR 0.83, 95%CI 0.77–0.90). Perinatal: No difference in admission to NICU, Apgar score and perinatal death.
41.	Janssen, 2001	The Care Environment	Intervention: Single room maternity unit where intrapartum and postpartum care are given in the same room with continuity of nursing care through labor, birth, and postpartum Where: Canada Population: Low-risk pregnant women Study Design: Intervention group compared to women historical control group Sample size: 430	PCC: More time with support people (*p* = 0.005), more time spent with newborn in room (*p* = 0.007), more privacy (*p* < 0.001), less noise (*p* < 0.001), more support from nurses (*p* < 0.001), Higher ratings for natural childbirth, making informed choices, having choices supported (*p* < 0.001). Increase in perceived knowledge (*p* < 0.001). Labor and Delivery: More comfort measures for pain in labor and postpartum pain (*p* < 0.001).
Person-centered Objective: Dignity				
42.	Abuya, 2015	Dignity	Intervention: Multilevel intervention aimed to address disrespect and abuse in childbirth, included engaging policymakers, training providers on respectful maternity care, and strengthening linkages between the facility and community for accountability and governance Where: Kenya Population: Postpartum women Study Design: Pre/post Sample size: 1369	PCC: Disrespect and abuse decreased from 20 to 13% (*p* < 0.004), some forms of disrespect and abuse decreased from 40 to 50%. Inappropriate detainment of women and infant in the facility declined from 8.0–0.8%. No difference in privacy violation and a small improvement confidentiality violation. No difference in abonnement.

**Table 3:** Detailed descriptions of included qualitative studies, organized by primary PCC objective

Study author and title		Type of intervention	Intervention details	Outcomes (Person-centered care (PCC), labor and delivery, perinatal, mental health)
Person-centered Objective: Autonomy				
1.	Brown, 2015	Autonomy	Systematic review and meta-analysis of RCTs of women-held case records, thematic analysis of qualitative data Sample size: 21	PCC Outcomes: Improved communication with providers, especially shared communication.
2.	De Koninck	Autonomy	Intervention: Continuity midwifery model implemented into birth centers that employed 3–6 midwives to provide care to one woman through prenatal, birth, and postpartum. Where: Canada Population: Pregnant women Study design: Intervention and matched controls Sample size: 10	PCC Outcomes: Improved communication with continuity midwifery model. Women reported holding back questions during rushed doctor visits. Continuity midwifery clients reported feeling respected and more humanized.
3.	Horey, 2015	Autonomy	Systematic review and meta-analysis of RCTs involving decision support for women with a prior caesarean, narrative synthesis of qualitative data. Sample size: 84	PCC Outcomes: Perceived benefits to having choices and information, but only information in appropriate context of risk and benefits.
4.	Walsh, 1999	Autonomy	Intervention: Continuity midwifery Where: England Population: Multiparous women Study Design: Ethnographic interviews Sample size: 10	PCC Outcomes: Women valued having continuity because it was easier to feel comfortable and ask questions. Felt empowered in labor.
Person-centered Objective: Supportive Care				
5.	Kildea, 2012	Supportive Care	Intervention: Specialist antenatal clinic for Australian Aboriginal and Torres Strait Islander women. Where: Australia Population: Australian Aboriginal and Torres Strait Islander women Study Design: A triangulation mixed-methods approach (including individual and focus group interviews; surveys) Sample size: 19	PCC Outcomes: Appreciated flexible drop-in schedule of the clinic.
6.	Stapleton, 2013	Supportive Care	Intervention: Specialty antenatal clinic for women from refugee backgrounds. Where: Australia Population: Women from refugee backgrounds Study Design: mixed-methods, data from hospital databases, a chart audit, surveys and interviews with service users, providers and stakeholders Sample size: 10	PCC Outcomes: Women appreciated the continuity model because it saved them time with translation; they didn’t have to repeat conversations.
Person-centered Objective: Social support				
7.	Hazard, 2009	Social Support	Intervention: Hispanic Labor Friends assisted women with communication with healthcare providers and emotional/physical Where: United States Population: Hispanic immigrant women Study Design: Descriptive qualitative inquiry Sample size: 21	PCC Outcomes: Women appreciated having the linguistic and cultural connection with Hispanic doulas. Women reported better informed consent.
8.	Herrman, 2012	Social Support	Intervention: Group ANC Where: United States Population: Pregnant women Study Design: A thematic and iterative analysis Sample size: 33	PCC Outcomes: Felt respected. Felt more informed by drawing from other women’s experiences.
9.	Risisky, 2013	Social Support	Intervention: Group ANC Where: United States Population: Pregnant women Study Design: Thematic analysis Sample size: 10	PCC Outcomes: Women reported richer information sharing in the group environment

### Results

#### Primary PCC objective#1: Autonomy (20 papers)

##### Autonomy: Interventions

The most common primary PCC objective was autonomy with 16 total interventions. Autonomy interventions fell broadly into two categories: labor and birth decisions and continuity midwifery. Specific labor and birth decision interventions implemented birth plans, open access to case notes, rigorous informed consent processes, and decision support. Of the 10 labor and birth decision interventions the largest proportion (4) tested some type of birth plan (Kuo [37], Lundgren [38], Martinez [39], Mehdizadeh [40]). The Horey [41] systematic review included three Randomized Control Trials (RCTs) of decision support for women who desired a vaginal birth after cesarean (VBAC), and Fraser [42] and Martin [43] examined specialty support clinics for women who desired a VBAC. The majority of interventions (8) connected women to specialized research staff (lay or clinical) to assist with individualized plans. Only two studies gave women access to information without additional assistance (Brown [44], O’Cathain [45]). Six manuscripts examined continuity midwifery care. Even though autonomy was the primary aim of these interventions, investigators stated multiple secondary person-centered objectives including supportive care, trust, dignity, privacy, and social support.

##### Autonomy: Outcomes

Eight of the ten labor and birth decision interventions measured autonomy, 2 out of 10 measured social support and supportive care, 1 of 10 measured the health facility environment, dignity, and communication. Generally, interventions either improved or made no difference to PCC outcomes, with the exception of Lundgren’s [38] birth plan experiment that showed a negative impact on dignity, communication, and supportive care. Seven of the studies looked at a labor and delivery outcomes, and found positive, negative, and null results. Regarding the negative outcomes, Brown [44] found women with access to case notes had more operative deliveries and cesarean sections, and Mehdizadeh [40] found more use of oxytocin. None of the studies that measured perinatal outcomes or maternal mental health found any impact. Five of the six continuity midwifery care interventions measured aspects of autonomy and all reported beneficial effects. Five of 6 measured trust by inquiring about the nature and extent of continuity with the known midwife. Three of 6 measured supportive care, communication, and dignity, while 1 of 6 measured privacy and the health facility environment. Continuous care with a midwife decreased obstetric interventions almost across the board. Only Gu [46] measured mental health and found no difference in levels of maternal anxiety.

##### Autonomy: Qualitative evaluations (4 papers)

Many of the qualitative evaluations concerning the PCC objective of autonomy confirmed the quantitative findings in that women generally gave positive reviews to decision support. Brown [44] found that women supported carrying their own pregnancy records to facilitate shared communication with providers. Horey [41] found four qualitative studies conducted concurrently with VBAC decision support trials; women with a prior cesarean perceived a sense of choice and gave positive evaluations to the information provided. However, some women reported that information about their options (VBAC or repeat cesarean) raised anxiety levels if the likelihood of certain risks was not included. In terms of feasibility, many women had to seek out additional support from research staff to use the decision tools.

Walsh [47] conducted ethnographic interviews with women (*N* = 10) in a continuity midwifery practice. Women placed great importance on their relationships with known midwives, and as a result felt more comfortable asking questions and felt that their concerns were validated. Home antenatal visits were well-reviewed because partners and children could be involved. While in labor at home, women appreciated having a known midwife and used expressions of “delight” to describe their labor experiences. This contrasted to women’s first hospital labors that felt de-humanized and lacked privacy. De Koninck [48] conducted a mixed-methods assessment of midwifery and medical care in Quebec and used open ended responses (*N* = 182) and interviews (*N* = 10) to contextualize the quantitative findings. Women described visits with doctors as “rushed” and “austere”. As a result, women reported feeling undervalued and held back questions. Clients of midwives “felt respected” and didn’t “feel like a number”. During labor women felt they had to be in a position suitable to the obstetrician, but with midwives, “I did not have to move for the midwife to be comfortable.” Women extended similar positive reviews to labor and delivery nurses, who they often found to be responsive to their needs [48] (Tables 4 and 5).

**Table 4:** How to interpret direction of outcomes in summary tables

Direction of outcome	PCC Outcomes	Labor and Delivery	Perinatal	Maternal Mental Health
Positive (+)	Improved the level of PCC	Decreased obstetric interventions	Decreased poor perinatal outcome (less pre-term birth, higher Apgar scores)	Improved mental health
Negative (−)	Decreased the level of PCC	Increased interventions	Increased poor perinatal outcome	Worsened mental health
No difference (=)	No change to the level of PCC	No change to interventions	No change to poor perinatal outcome	No change to mental health

**Table 5:**
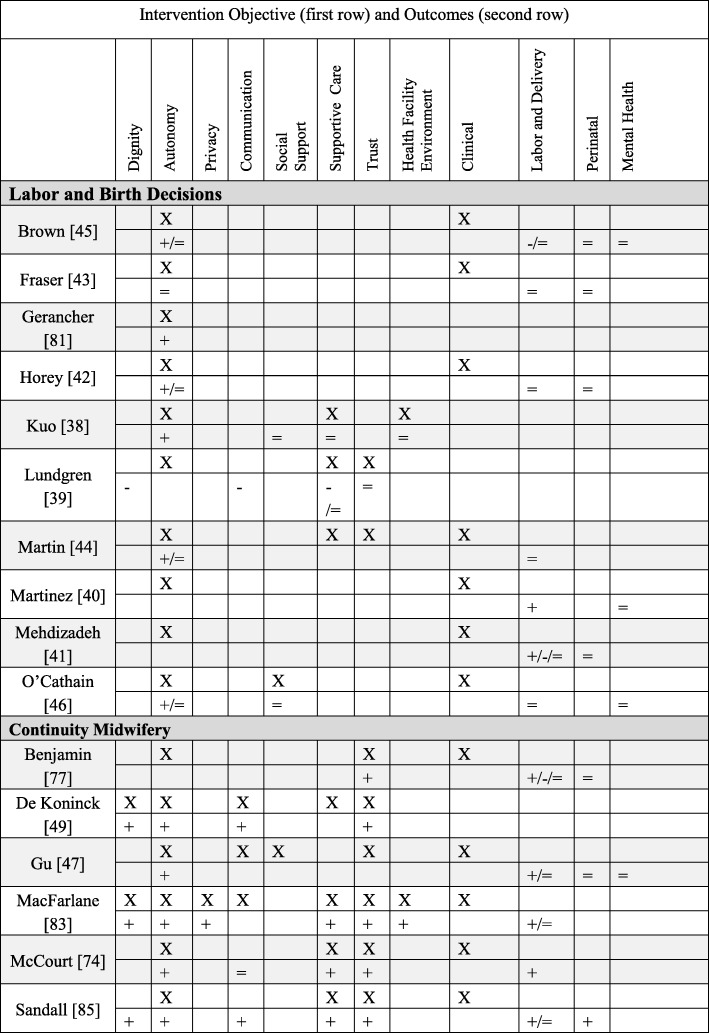
Primary PCC Objective: Autonomy

#### Primary PCC objective#2: Supportive care (17 papers)

##### Supportive care: Interventions

The second most common set of interventions identified in this review concerned supportive care (*N* = 15). These interventions fell broadly into two categories: enhanced prenatal care for at-risk women and psychological support. A total of 9 studies targeted at-risk women, including adolescents (Grassley [49]), low-income and/or ethnic minority women (El-Mohandes [50], Harris [51], Kildea [52]), and women at risk for pre-term birth (Hodnett [53], Mason [54], Newman [55], Panaretto [56]). Generally, this group of interventions aimed to optimize access to quality prenatal care, in order to decrease the effects of socio-medical risk factors that influence adverse pregnancy outcomes. Another 6 studies in the supportive care category sought to intervene upon the woman’s psychological state, especially as it related to anxiety or fear of childbirth, her level of mindfulness, and her coping skills.

##### Supportive care: Outcomes

While the stated objective was to provide supportive care to at-risk women, no study actually measured supportive care. Of the three studies focused on low-income or ethnic minority women, only one inquired about the level of culturally sensitive-care women received (Kildea [52]). Two of 6 studies inquired about various aspects of at-risk women’s autonomy. The majority of trials focused on at-risk women sought to decrease pre-term birth and found positive or null results. Those that measured clinical outcomes (epidural, cesarean, planned VBAC) generally also had positive or null results. Compared to the at-risk women, psychological interventions consistently measured person-centered outcomes, including support (3/6) and autonomy (3/6). Many also explored the impact on mental health (5/6) using validated scales for depression, anxiety, and post-traumatic stress disorder (PTSD). Results on PCC measures were mixed, with Ryding [57] finding that women in the intervention group reported a more frightening experience in childbirth and more post-traumatic stress. Clinical outcomes measured were generally positive (more spontaneous vaginal births, shorter labor lengths). Perinatal outcomes were not frequently explored, and those that did found no impact.

##### Supportive care: Qualitative evaluations (2 papers)

Kildea [52] conducted a mixed methods analysis of a specialized clinic for ethnic minority women. In face-to-face interviews women gave positive reviews to the continuity of care model, as women liked not having to repeat information with providers. Women reported that it was more important that the antenatal provider listened to them rather than share the same cultural identity. Women also appreciated the flexible drop-in appointment system and the proximity of the clinic to the labor ward, which helped partners/family know where to take the women when labor started. Some women reported that the waiting room could be crowded and lacked privacy. While in labor, women wanted to maintain physical modesty and privacy by limiting the number of hospital staff. Finally, they feared that people entering the room indicated a problem with the labor or baby.

Stapleton [58] conducted 4 focus groups with refugee women about their experiences with a specialized antenatal clinic. Refugee women also appreciated the continuity model of care because they did not have to repeat traumatic histories. Continuity allowed for more efficient use of interpreters; however, a challenge was clients using interpreters as sources of clinical information. Geographic distance created barriers to access, either because language difficulties arose on public transit or women because women had to rely on husbands for transportation. Refugee women were accustomed to having female kin support them in labor and reported feeling isolated in the new Australian context (Table 6).

**Table 6:**
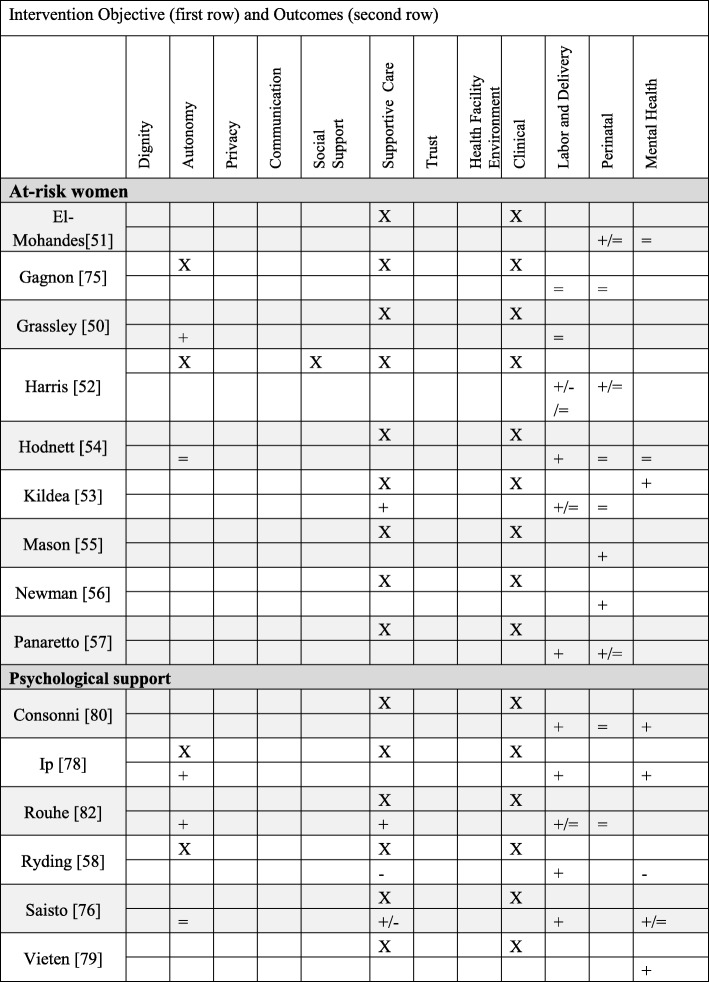
Primary PCC objective: Supportive Care

#### Primary PCC objective#3: Social support (11 papers)

##### Social support: Interventions

The third group of interventions sought to increase support for women during pregnancy and birth care through involvement of male partners and/or continuous labor support, or group prenatal care. Through these interventions male partners were invited to be more involved in care, or women were provided with the continuous support of a doula during labor and birth. Group prenatal care interventions encouraged women to connect and support each other outside the confines of individual prenatal visits.

##### Social support: Outcomes

While the expressed objective of male partner and continuous labor support interventions was social support, only Gungor [59] and Kunene [60] actually measured support, both with positive outcomes. Three of the male partner/continuous support studies measured autonomy (Gungor [59], Hodnett [61], Mullany [62]), all with positive effects. Results were mixed for clinical outcomes, with some evidence of positive impact on labor and delivery (Hodnett [61]). However, other studies found no impact on pain meds, obstetric interventions, or type of delivery (Mullany [62], Gungor [59], Gruber [63]). Two papers looked at perinatal outcomes (Hodnett [61]; Gruber [63]) with positive or null results. None of the group prenatal care studies measured social support. Two of the three group prenatal care studies measured autonomy with positive or null results. Results were mixed for clinical outcomes, with some significant (Barr [64]) and some null findings (Catling [65]). Similarly, results were mixed for perinatal outcomes (lower preterm birth for Barr [64] and no difference for Catling [65]). Only Catling measured maternal mental health and found no differences in depression with group prenatal care.

##### Social support: Qualitative results (3 papers)

Herrman [66] explored the strengths and weaknesses of group antenatal care by conducting 5 focus groups. Women felt respected in the group environment, drew on the knowledge of the other mothers in the room, and reported a greater sense of capability to become mothers. Risisky [67] conducted 3 focus groups of group antenatal care during which women reported appreciating the rich conversations created by women sharing experiences together. This helped women feel more empowered as decision makers. Women appreciated having partners attend the group prenatal sessions, as this helped partners become more effective sources of support. Even though few quantitative studies measured social support, social support was a prominent theme in the qualitative evaluations.

Hazard [68] interviewed Spanish-speaking Hispanic women to evaluate a culturally-sensitive program of labor support compared to women who received standard care. Women who received the intervention appreciated having the cultural and social support from trained Hispanic doulas, demonstrated increased use of healthcare resources, reported enhanced quality of informed consent, and fewer language barriers with providers. As a whole the qualitative data did not address clinical outcomes with the exception of Hazard [68], who reported that intervention women exhibited more care-seeking behaviors (Table 7).

**Table 7:**
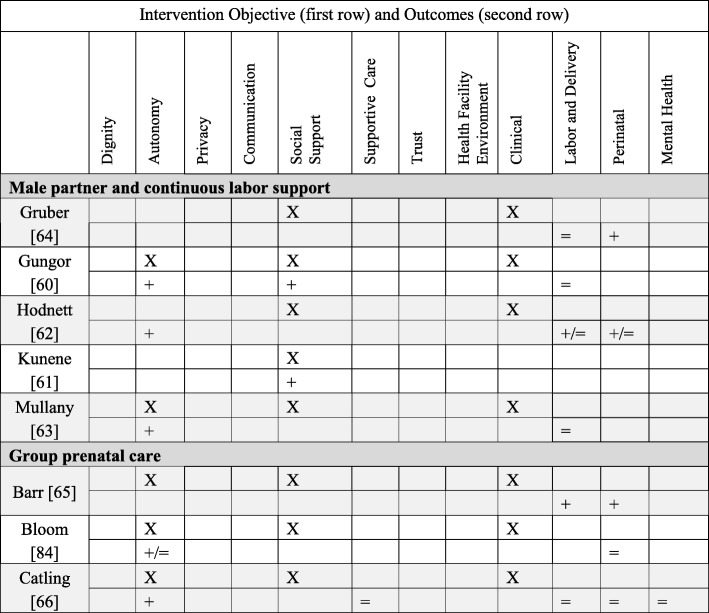
Primary PCC Objective: Social Support

#### Primary PCC objective#4: The health facility environment (2 papers)

##### The health facility environment: Interventions

Two studies examined the health facility environment through alternative birth sites (Hodnett [69]) and a new physical organization for a labor room (Janssen [70]). These interventions also intended to impact trust and clinical outcomes.

##### The health facility environment: Outcomes

Janssen [70] measured women’s perceptions of the new physical space, which were positive; Hodnett [69] did not measure women’s perceptions of the alternative health environment. Regarding other PCC outcomes, Hodnett only measured autonomy, with a positive impact. Janssen found positive impacts on dignity, autonomy, privacy, social support, supportive care, and the health facility environment. Both studies found positive impacts on clinical outcomes, with lower rates of epidurals, labor augmentation, and episiotomy and higher rates of vaginal birth (Hodnett) and women using more comfort measures for pain (Janssen). Hodnett found a reduction in low Apgar scores. Neither study measured maternal mental health outcomes.

#### Primary PCC objective#5: Dignity (1 paper)

##### Dignity: Interventions

One intervention (Abuya [71]) included in this review utilized the current framework of respectful maternity care. We categorized the primary objective of Abuya as increasing dignity by decreasing mistreatment of women, although the intervention took a multi-pronged approach and addressed several secondary PCC objectives.

##### Dignity: Outcomes

Abuya found a positive impact on dignity (decreased disrespect and abuse of women), an increase in women’s autonomy, and no difference in supportive care and privacy. No clinical outcomes were measured (Tables 8 and 9).

**Table 8:**
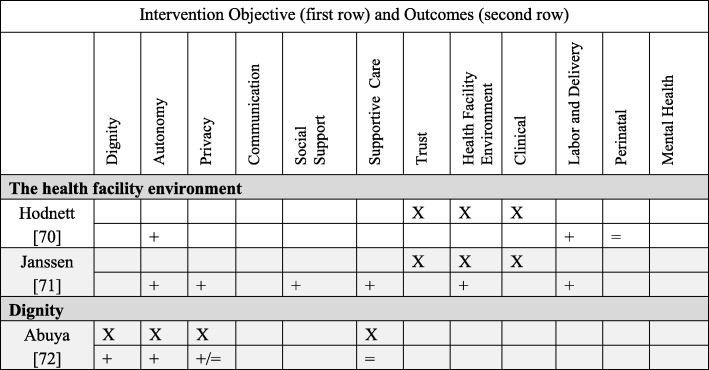
Primary PCC Objective: The Health Facility Environment and Dignity

**Table 9:**
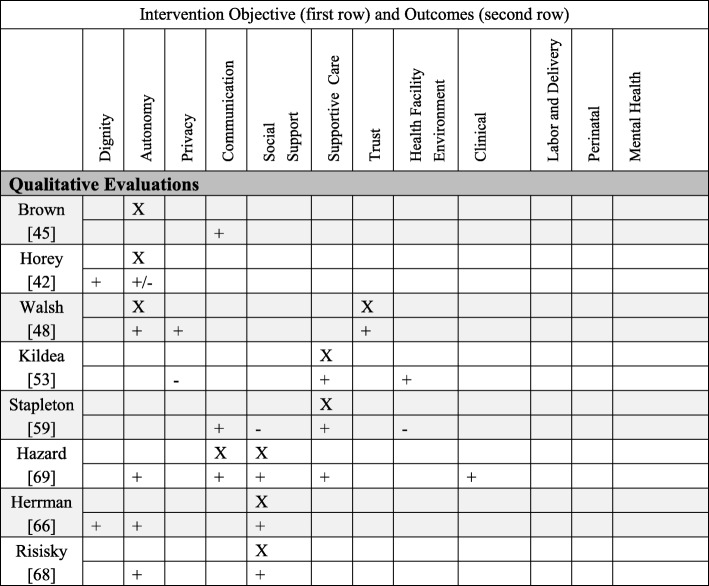
Primary PCC Objectives: Qualitative Evaluations

## Discussion

We conducted the first systematic review of person-centered care interventions in birth facilities using a current and comprehensive framework for PCC. We found that since the 1990s the absolute number of PCC delivery interventions has increased. We found that applying a current PCC framework was feasible and applicable to multiple prior interventions, covering five primary PCC objectives (autonomy, supportive care, social support, the health facility environment, and dignity). Past PCC interventions attempted to empower and support pregnant women to a variety of ends, usually to decrease inappropriate obstetric interventions, improve perinatal outcomes, to directly impact maternal mental health, or decrease disrespect and abuse. We found very few examples of harm caused by a person-centered intervention, and many examples of either null or positive effects.

Given the contextual nature of person-centered objectives, using the mixed-methods systematic review allowed us to examine the contour and correlations between PCC objectives and PCC outcomes. Within our own consistent use of PCC categories, we found that PCC objectives frequently did not overlap with PCC outcomes. For instance, while supportive care was the explicit goal for at-risk women, none in this group of interventions measured supportive care. At the other end of the spectrum, some researchers found improvements in PCC outcomes that were not stated anywhere in the intervention’s objectives.

Building on Sudhinaraset et al.’s framework [29], our review highlights several gaps in the PCC intervention literature. While we did find numerous studies that explored the relationship between PCC and the provision of care, we found only one intervention that linked the health facility to changes in the health system, gender and violence norms, or community care-seeking behaviors. Abuya et al linked the mistreatment of women in the facility to “community accountability and governance” [71]. Also, while we did find a number of interventions designed to improve perinatal outcomes for at-risk women, none of these interventions measured individual perceptions of discrimination, nor did these interventions attempt to link the facility to external accountability mechanisms nor to structural interventions to transform systemic discrimination [72].

This review raises questions about the theoretical coherence of PCC interventions, in terms of how faithfully researchers should match objectives and outcomes, which combinations of domains might result in the greatest benefit (or rarely, harm), and the relationship between PCC objectives and clinical outcomes. Thus, our review builds on a growing body of evidence as to the heterogeneous approaches to measuring and intervening upon women’s experiences of evidence-based, quality maternity care [5, 17]. By tying together past interventions with the newer frameworks around respectful maternity care, we have demonstrated a longer tradition of person- centered objectives in maternity care. Future interventions can and should draw on the rich literature related to decision-support, continuity midwifery, group prenatal care, and alternative birth sites.

### Limitations

There are several limitations to this systematic review. First, the nature of our search terms and exclusion strategy lead to many papers from low-resource settings being excluded and thus the majority of papers are from high-resource settings. While there is a longer history of interventions on person-centered care from the developed world and subsequently more literature, it is unclear if these findings are as relevant to a developing world setting. As mentioned above, women in different settings may desire different aspects of person-centeredness, so interventions in one setting may not be relevant in another. Relatedly, while our definition of PCC was based on findings from around the globe, our domains might not appropriately represent all women’s expectations. Furthermore, we limited our search to interventions in facilities and to outcomes measured post-delivery. There are many exciting person-centered interventions that were exclusively based in the community, prenatal or post-natal settings. These diverse PCC interventions deserve attention and critical review. Finally, we found that the qualitative literature was very narrow based on our search terms; we recommend that a future mixed-methods systematic review use broader search terms, especially to identify relevant studies that were not directly attached to an intervention.

## Conclusions

We conducted a systematic review of 47 past person-centered care interventions in birth facilities to map the range of their PCC objectives and to examine their range of impacts. We recommend that future interventions be more explicit about how and why certain PCC objectives are chosen and to measure PCC outcomes that match the stated PCC objectives. Matching objectives to outcomes will further our understanding of the mechanisms that underlie PCC interventions. Finally, nesting the PCC objectives within a model of care has been a consistent approach in the literature and should be strongly considered for future interventions. How to link facilities to external accountability mechanisms and to the community are underexplored approahes  that could be promising for future interventions.

## Additional files

Additional file 1: Definitions of PCC Objectives. (DOCX 30 kb)

Additional file 2: Search Strategy. (DOCX 13 kb)

Additional file 3: Risk of Bias. (DOCX 15 kb)

## Abbreviations

PCC: Person-centered care
PTSD: Post-traumatic stress disorder
RCT: Randomized control trial
RMC: Respectful maternity care
VBAC: Vaginal birth after cesarean
