# Supplementary material for: A systematic review of person-centered care interventions to improve quality of facility-based delivery
Source: Reprod Health. 2018 Oct 10;15:169. doi: 10.1186/s12978-018-0588-2 (PMC6180507; doi:10.1186/s12978-018-0588-2)
Supplement: Supplementary file 1 — Definitions of PCC Objectives. (DOCX 30 kb) [file 12978_2018_588_MOESM1_ESM.docx]

# Table S1: Person centered objectives and their definitions, adapted from Sudhinaraset et al (2017)

| Dignity | Dignity refers to the ability of women to receive care in a respectful and caring setting. It captures the typologies of physical and verbal abuse from the literature on mistreatment of women during labor and delivery, as well as receipt of care in a timely manner. |
| --- | --- |
| Autonomy | Autonomy refers to respecting women’s views of what is appropriate care and allowing her to make informed choices. This includes providing consented care. An example of a measure for autonomy is whether women feel involved in decision-making about their care and whether their permission is sought before treatments. |
| Privacy/Confidentiality | Privacy/Confidentiality concern the environment in which care is provided and the concept of privileged communication and confidentiality of medical records. An example is whether women feel others who are not involved in their care could hear information about their care or could see them during physical examinations. |
| Communication | Communication refers to providers clearly explaining to women and family the nature of their condition, details of treatment, and available treatment options. An example is whether providers clearly explain to women their conditions to them and the purpose of treatments as well as any side effects of treatments. |
| Social Support | Social support reflects the extent to which women have access to their family and friends when receiving care. It also includes their right to receive food and other consumables from family where deemed appropriate. |
| Supportive Care | Supportive care refers to providing care in a compassionate manner. It includes women’s perceptions of how providers respond to them when they need more help. It also captures abandonment or denial of care. |
| Trust | Trust captures how women assess their relationships with providers. Here, measures include whether women feel providers tell them the truth about their care and whether they have confidence in the competence of their providers. |
| Stigma/Discrimination | This captures the ability to receive dignified care irrespective of one’s status or other personal attributes. Measures here include whether women feel they are treated differently based on their age, wealth, or ethnicity. |
| Health Facility Environment | This refers to the quality of the facility, including having basic amenities and the extent to which a health facility offers a welcoming and pleasant environment. Examples include clean surroundings and enough space in waiting rooms and wards. |

# Search Strategy

## Final Pubmed Keywords:

((("Maternal Health Services"[majr] OR "maternal health"[tiab] OR "maternal welfare"[MeSH] OR "maternal welfare"[tiab] OR antenatal[tiab] OR "ante natal"[tiab] OR perinatal[tiab] OR "peri natal"[tiab] OR postnatal[tiab] OR "post natal"[tiab] OR postpartum[tiab] OR "post partum"[tiab] OR intrapartum[tiab] OR "intra partum"[tiab] OR "maternal mortality"[tiab] OR "maternal death"[tiab] OR "maternal morbidity"[tiab] OR "maternal mortality rate"[tiab] OR "maternal complications"[tiab] OR "obstetric delivery"[tiab] OR "obstetric deliveries"[tiab] OR "delivery, obstetric"[Mesh] OR "obstetric labor"[tiab] OR "cesarean section"[MeSH] OR "caesarean section"[tiab] OR "vaginal birth"[tiab] OR pregnancy[tiab] OR childbirth[tiab] OR parturition[tiab] OR birth[tiab] OR births[tiab] OR "live birth"[tiab] OR "home childbirth"[tiab] OR "traditional birth attendant"[tiab] OR "skilled birth attendant"[tiab] OR doula[tiab] OR doulas[tiab] OR midwife[tiab] OR midwives[tiab]) AND ("Human Rights"[MeSH] OR "patient-centered care"[MeSH Terms] OR "patient-centered care"[tiab] OR "patient-centred care"[tiab] OR "woman centered care"[tiab] OR "woman centred"[tiab] OR "person centered care"[tiab] OR "person centred care"[tiab] OR "client centered care"[tiab] OR "client centred care"[tiab] OR communication[tiab] OR communicate[tiab] OR respect[tiab] OR disrespect[tiab] OR disrespectful[tiab] OR dignity[tiab] OR stigma[tiab] OR neglect[tiab] OR mistreatment[tiab] OR "emotional support"[tiab] OR "experience of care"[tiab] OR abuse[tiab] OR privacy[tiab] OR "perceived quality"[tiab] OR "patient satisfaction"[tiab] OR "healthcare quality"[tiab] OR "cultural competence"[tiab] OR "clinical competence"[tiab] OR "informed choice"[tiab] OR counseling[tiab] OR "patient provider interaction"[tiab] OR "provider responsiveness"[tiab] OR "patient participation"[tiab] OR "patient involvement"[tiab] OR "patient empowerment"[tiab] OR "patient engagement"[tiab] OR "patient safety"[tiab] OR "quality of health care"[tiab] OR "shared decision making"[tiab] OR "centering pregnancy"[tiab] OR "birth plan"[tiab] OR "patient choice"[tiab] OR "patient autonomy"[tiab] OR "informed consent"[tiab]) AND (intervention[tiab] OR evaluation[tiab] OR program[tiab] OR "program evaluation"[MeSH] OR "program evaluation"[tiab])))

## Gray literature google search of multi-lateral organizations, limited to the first 100 hits ordered by relevance:

United Nations Fund for Population, United Nations Development Fund for Women, African Development Bank, Asian Development Bank, United Kingdom’s Department for International Development, United States Agency for International Development, World Bank, World Health Organization Institute for Healthcare Improvement, The International Committee for Research on Women, Population Council, The Global Fund for Women, The Hewlett Foundation, The Packard Foundation, The Guttmacher Institute, ANSIRH, Ipas, Ibis, Gates Foundation, Jhpiego, Engender Health, IPPF, Marie Stopes International, Population Services International

# Figure S1. Summary of risk of bias assessment of included studies

# Table S2. Summary of critical appraisal of all included qualitative studies

| **Summary critical appraisal of all included qualitative studies** | *Yes* | *Can't tell* | *No* |
| --- | --- | --- | --- |
| **Clear statement of study aims** | 100% | 0% | 0% |
| **Appropriate qualitative methodology** | 100% | 0% | 0% |
| **Appropriate research design** | 100% | 0% | 0% |
| **Appropriate recruitment strategy** | 100% | 0% | 0% |
| **Appropriate data collection method** | 100% | 0% | 0% |
| **Consideration of researcher relationship** | 71% | 0% | 29% |
| **Consideration of ethical issues** | 100% | 0% | 0% |
| **Rigorous data analysis** | 86% | 0% | 14% |
| **Clear statement of findings** | 100% | 0% | 0% |
