# Supplementary material for: A systematic review of person-centered care interventions to improve quality of facility-based delivery
Source: Reprod Health. 2018 Oct 10;15:169. doi: 10.1186/s12978-018-0588-2 (PMC6180507; doi:10.1186/s12978-018-0588-2)
Supplement: Supplementary file 2 — Search Strategy. (DOCX 13 kb) [file 12978_2018_588_MOESM2_ESM.docx]

# Search Strategy

## Final Pubmed Keywords:

((("Maternal Health Services"[majr] OR "maternal health"[tiab] OR "maternal welfare"[MeSH] OR "maternal welfare"[tiab] OR antenatal[tiab] OR "ante natal"[tiab] OR perinatal[tiab] OR "peri natal"[tiab] OR postnatal[tiab] OR "post natal"[tiab] OR postpartum[tiab] OR "post partum"[tiab] OR intrapartum[tiab] OR "intra partum"[tiab] OR "maternal mortality"[tiab] OR "maternal death"[tiab] OR "maternal morbidity"[tiab] OR "maternal mortality rate"[tiab] OR "maternal complications"[tiab] OR "obstetric delivery"[tiab] OR "obstetric deliveries"[tiab] OR "delivery, obstetric"[Mesh] OR "obstetric labor"[tiab] OR "cesarean section"[MeSH] OR "caesarean section"[tiab] OR "vaginal birth"[tiab] OR pregnancy[tiab] OR childbirth[tiab] OR parturition[tiab] OR birth[tiab] OR births[tiab] OR "live birth"[tiab] OR "home childbirth"[tiab] OR "traditional birth attendant"[tiab] OR "skilled birth attendant"[tiab] OR doula[tiab] OR doulas[tiab] OR midwife[tiab] OR midwives[tiab]) AND ("Human Rights"[MeSH] OR "patient-centered care"[MeSH Terms] OR "patient-centered care"[tiab] OR "patient-centred care"[tiab] OR "woman centered care"[tiab] OR "woman centred"[tiab] OR "person centered care"[tiab] OR "person centred care"[tiab] OR "client centered care"[tiab] OR "client centred care"[tiab] OR communication[tiab] OR communicate[tiab] OR respect[tiab] OR disrespect[tiab] OR disrespectful[tiab] OR dignity[tiab] OR stigma[tiab] OR neglect[tiab] OR mistreatment[tiab] OR "emotional support"[tiab] OR "experience of care"[tiab] OR abuse[tiab] OR privacy[tiab] OR "perceived quality"[tiab] OR "patient satisfaction"[tiab] OR "healthcare quality"[tiab] OR "cultural competence"[tiab] OR "clinical competence"[tiab] OR "informed choice"[tiab] OR counseling[tiab] OR "patient provider interaction"[tiab] OR "provider responsiveness"[tiab] OR "patient participation"[tiab] OR "patient involvement"[tiab] OR "patient empowerment"[tiab] OR "patient engagement"[tiab] OR "patient safety"[tiab] OR "quality of health care"[tiab] OR "shared decision making"[tiab] OR "centering pregnancy"[tiab] OR "birth plan"[tiab] OR "patient choice"[tiab] OR "patient autonomy"[tiab] OR "informed consent"[tiab]) AND (intervention[tiab] OR evaluation[tiab] OR program[tiab] OR "program evaluation"[MeSH] OR "program evaluation"[tiab])))

## Gray literature google search of multi-lateral organizations, limited to the first 100 hits ordered by relevance:

United Nations Fund for Population, United Nations Development Fund for Women, African Development Bank, Asian Development Bank, United Kingdom’s Department for International Development, United States Agency for International Development, World Bank, World Health Organization Institute for Healthcare Improvement, The International Committee for Research on Women, Population Council, The Global Fund for Women, The Hewlett Foundation, The Packard Foundation, The Guttmacher Institute, ANSIRH, Ipas, Ibis, Gates Foundation, Jhpiego, Engender Health, IPPF, Marie Stopes International, Population Services International
