# Supplementary material for: A systematic review of person-centered care interventions to improve quality of facility-based delivery
Source: Reprod Health. 2018 Oct 10;15:169. doi: 10.1186/s12978-018-0588-2 (PMC6180507; doi:10.1186/s12978-018-0588-2)
Supplement: Supplementary file 3 — Risk of Bias. (DOCX 15 kb) [file 12978_2018_588_MOESM3_ESM.docx]

# Figure 1. Summary of risk of bias assessment of included studies

# Table 2. Summary of critical appraisal of all included qualitative studies

| **Summary critical appraisal of all included qualitative studies** | *Yes* | *Can't tell* | *No* |
| --- | --- | --- | --- |
| **Clear statement of study aims** | 100% | 0% | 0% |
| **Appropriate qualitative methodology** | 100% | 0% | 0% |
| **Appropriate research design** | 100% | 0% | 0% |
| **Appropriate recruitment strategy** | 100% | 0% | 0% |
| **Appropriate data collection method** | 100% | 0% | 0% |
| **Consideration of researcher relationship** | 71% | 0% | 29% |
| **Consideration of ethical issues** | 100% | 0% | 0% |
| **Rigorous data analysis** | 86% | 0% | 14% |
| **Clear statement of findings** | 100% | 0% | 0% |
